# Disc inflammation and intercellular communication in shaping the immune microenvironment of intervertebral disc degeneration

**DOI:** 10.3389/fimmu.2025.1719293

**Published:** 2025-11-19

**Authors:** Mengcheng Wei, Kanghua Zhu

**Affiliations:** Department of Pain, The Sixth Hospital of Wuhan, Affiliated Hospital of Jianghan University, Wuhan, China

**Keywords:** intervertebral disc degeneration, intercellular communication, machine learning, artificial neural network, immune differences, biomarkers

## Abstract

**Background:**

Intervertebral disc degeneration (IDD) is a major cause of low back pain, significantly affecting the quality of life of elderly individuals worldwide. Its pathogenesis is complex, involving extracellular matrix degradation, inflammatory responses, and immune regulation.

**Methods:**

This study integrated multiple transcriptomic and single-cell RNA-seq datasets, and systematically identified and validated key IDD-related genes and their immune regulatory roles through differential gene analysis, GO/KEGG enrichment, PPI network construction, ANN, LASSO, random forest, SVM-RFE, SHAP, Mendelian Randomization, as well as immune cell infiltration and CellChat analyses. The identified candidate genes were further validated experimentally using Western blot, confirming their expression patterns and potential as diagnostic biomarkers.

**Results:**

By integrating bioinformatics with in vitro validation, this study identified three key biomarkers associated with IDD: MMP9, HPGD, and UCHL1. SHAP analysis demonstrated that these genes make significant contributions to the diagnostic model, primarily participating in immune regulation and inflammatory responses. Functional enrichment analysis indicated their involvement in signaling pathways such as IL-17, TNF, and MAPK. Correlation and differential analyses of immune cells showed that γδ T cells exhibited significant changes across all three genes, while other immune cell types, such as CD4⁺ T cells, displayed differences in the remaining biomarkers. Single-cell analysis further revealed that the MIF signaling pathway plays a key role in the interactions between nucleus pulposus cells and immune cells.

**Conclusion:**

These findings provide new insights into the molecular and immune mechanisms of IDD and offer potential targets for diagnosis and therapy.

## 1 Introduction

Lower back pain (LBP) is a prevalent musculoskeletal disorder and a major cause of disability among the elderly worldwide (1, 2). Numerous studies have identified intervertebral disc degeneration (IDD) as the primary pathological factor underlying LBP (3, 4). In adults, the intervertebral disc (IVD) is an avascular tissue composed of three distinct parts: the nucleus pulposus (NP) at the center, the annulus fibrosus (AF) at the periphery, and the cartilaginous endplates (CEP) on the top and bottom surfaces. Each region contains specific cell populations with specialized functions. The initiation and progression of IDD are driven by multiple factors, including aging, oxidative stress, inflammation, and abnormal mechanical loading. Hallmark pathological changes include NP degeneration, AF rupture, and CEP calcification (5, 6). Despite these observations, the precise molecular mechanisms underlying disc degeneration remain unclear.

Epidemiological studies have shown that the prevalence of IDD is increasing globally, making it an important public health issue that imposes significant social and economic burdens (7). The etiological mechanisms of IDD are highly complex, involving genetic predisposition, immune-inflammatory responses, extracellular matrix (ECM) metabolic imbalance, as well as abnormalities in apoptosis and autophagy (8–10). For instance, aberrant activation of matrix metalloproteinases (MMPs) and cathepsins has been reported to promote ECM degradation and disrupt NP collagen homeostasis, thereby accelerating disc degeneration (11). In addition, infiltration of immune cells such as macrophages and T lymphocytes can aggravate ECM breakdown and cell death by releasing pro-inflammatory cytokines, including TNF-α and IL-1β, thus further driving the progression of IDD (12).

Advances in high-throughput techniques and bioinformatics in recent years have enabled differential expression analysis, WGCNA, and machine learning to identify crucial genes and signaling pathways linked to IDD (13). Building on this, the present study integrates multiple bioinformatics strategies to systematically elucidate the molecular mechanisms of IDD. Network pharmacology combines computational modeling with bioinformatics to establish drug–target and signaling networks, thereby facilitating drug discovery and enhancing the precision of therapeutic prediction (14). Mendelian randomization (MR) uses genetic variants as instrumental variables to reduce confounding and evaluate possible causal relationships between exposures and diseases (15). Progress in high-throughput technologies and bioinformatics has greatly advanced insights into intercellular communication within pathological tissues. Machine learning (ML) enables the analysis of large-scale, complex datasets and the identification of latent biological patterns (16, 17); however, its inherent “black-box” problem constrains its clinical translation (18). To overcome this limitation, the SHapley Additive exPlanations (SHAP) method, derived from game theory, provides an interpretable approach that quantifies the influence of individual features on model predictions (19, 20).

Our findings indicate that patients with IDD display distinct gene expression profiles compared with healthy controls. These genes are predominantly enriched in inflammatory and immune regulatory pathways, in line with earlier reports (21). Immune infiltration analysis further highlighted dynamic shifts in immune cell composition as a hallmark of IDD pathogenesis. We suggest that aberrant gene expression may serve as a trigger for IDD, exerting its effects on immune cells through various mechanisms and ultimately driving marked alterations in their expression patterns and proportions. First, dysregulated gene expression in IDD may lead to inappropriate activation or inhibition of pathological signaling pathways, causing substantial secretion of inflammatory factors. Second, the heightened expression of these mediators interferes with immune homeostasis (22). Ultimately, the dysregulated immune response results in differential expression of immune cells within the body (23). In conclusion, abnormal gene expression is crucial to the onset and development of IDD. To clarify this mechanism, we integrated multichip analysis, PPI network construction, ANNs, and machine learning to screen key differentially expressed genes. Subsequently, SHAP modeling and eQTL-based MR analysis were applied to assess their diagnostic relevance. We further examined immune cell correlations and expression differences across cell subsets, and incorporated single-cell sequencing to investigate intercellular communication within the immune microenvironment, thereby revealing its role in IDD progression.

## 2 Materials and methods

### 2.1 Data sources

We retrieved IDD–related datasets (GSE124272, GSE150408, and GSE153761) from the GEO database (https://www.ncbi.nlm.nih.gov/geo/). These datasets included transcriptomic profiles from 28 patients with IDD and 28 healthy controls. In addition, we obtained single-cell transcriptomic data from three IDD patients (GSM7831817, GSM7831818, GSM7831819) in the GSE244889 dataset to investigate the cellular composition and molecular characteristics of IDD tissues. The detailed information of the datasets is provided in **Supplementary Table S1**. Data preprocessing was carried out using R software (version 4.3.1). To minimize batch effects and ensure comparability between groups, we applied the “limma” (version 3.62.2), “pheatmap” (version 1.0.12) and “ggplot2” (version 3.5.1) packages. Differentially expressed genes (DEGs) were visualized with heatmaps generated by “pheatmap” (version 1.0.12) and volcano plots produced by “ggplot2”. All expression data were log2-transformed prior to analysis to improve reliability.

### 2.2 Identification of differentially expressed genes

After standardizing the dataset, we applied the SVA package to correct for batch effects. Subsequently, principal component analysis (PCA) was performed to evaluate the effectiveness of the standardization process. The “limma” R package was employed for preprocessing gene expression data, followed by differential expression analysis between breast cancer and control groups. Genes with |log_2_FC| ≥ 1 and adjusted P ≤ 0.05 (Benjamini–Hochberg) were considered DEGs.

### 2.3 KEGG and GO analysis

The “clusterProfiler” (version 4.14.4) R package was used for GO and KEGG enrichment analyses to characterize the biological functions and pathways linked to the DEGs.

### 2.4 Artificial neural network

ANNs are widely used for predictive modeling due to their ability to capture nonlinear relationships in high-dimensional data. A typical ANN consists of an input layer, hidden layer(s), and an output layer, enabling efficient data processing and prediction (24). In this study, correlation analysis was performed in R, and the “neuralnet” (version 1.44.2) and “NeuralNetTools” (version 1.5.3) packages were used for model construction and visualization.

### 2.5 Machine learning algorithms

LASSO analysis was performed using the glmnet package with 10-fold cross-validation to optimize the penalty parameter, and the random seed was set to 123 to ensure reproducibility. A multilayer perceptron (MLP) neural network model was constructed using the MLP Classifier from Scikit-learn and trained on the transposed gene expression data. The model consisted of two hidden layers with 50 and 15 nodes, respectively, with a maximum of 1000 iterations, and the random seed was fixed at 42 to ensure reproducibility. Support Vector Machine Recursive Feature Elimination (SVM-RFE) was performed using an SVM model with a radial basis function (RBF) kernel, with the random seed set to 123. Gene features ranging from 2 to 40 were screened via cross-validation, and the gene set corresponding to the minimum RMSE was selected. Additionally, the randomForest package (version 4.7-1.4) was used to identify genes with an importance score greater than 2, with the random seed set to 123456. Finally, the intersection of results from LASSO, random forest (RF), SVM-RFE, and neural network models was used to identify key marker genes in IDD patients for further analysis and validation.

### 2.6 SHAP methodology

To enhance the interpretability of the machine learning models, we employed the SHAP method, a game theory–based approach that quantitatively evaluates the contribution of each feature to the model’s predictions, thereby clarifying its positive or negative impact on the outcomes. Based on the selected predictive factors, we constructed ten machine learning models, including RLS, RF, DTS, SVM, logistic regression, KNN, XGBoost, GBM, neural network, and GlmBoost. The optimal model was chosen as the primary framework for this study. To improve interpretability, SHAP were employed to evaluate feature contributions, offering insights into potential biological mechanisms.

### 2.7 MR analysis

Mendelian Randomization (MR) analysis uses genetic variants as instrumental variables to simulate a natural randomized experiment, thereby inferring the causal relationship between exposures and outcomes. We performed MR analysis using the inverse variance weighted (IVW) method as the primary estimator. Single nucleotide polymorphisms (SNPs) significantly associated with the exposure (P < 5 × 10^−6^) were pruned for linkage disequilibrium (R² < 0.001, distance > 10,000 kb) to ensure independence. Instrument strength was assessed by calculating F-statistics, with all values >10, indicating no weak instrument bias. Horizontal pleiotropy was evaluated using the MR-Egger intercept test (P > 0.05) and further examined by MR-PRESSO to detect and remove potential outliers. **Supplementary Figure S1** provides a schematic illustration of the MR analysis.

### 2.8 Infiltration analysis

Immune cell abundance was estimated by deconvolving immune cell subtype expression matrices using CIBERSORT in R. This method leverages 22 common immune cell types to analyze the immune composition of samples.

### 2.9 Single-cell analysis

scRNA-seq data were quality-controlled by filtering out low-quality cells (<50 genes, >15% mitochondrial reads) and removing erythrocyte and ribosomal contamination. The data were log-normalized, integrated using Seurat, and then scaled. Batch effects were corrected using Harmony to minimize technical variation between samples. Clustering was performed on the top 20 Harmony-corrected principal components: a nearest-neighbor graph was constructed, and clusters were identified using the Louvain algorithm with a resolution of 0.6. Cell types were annotated with SingleR (version 2.8.0). t-SNE was used to visualize the clustering results, with a perplexity of 30 and a maximum of 1000 iterations to preserve both local and global structure. Intercellular communication in IDD was analyzed using CellChat (version 1.6.1).

### 2.10 Cell culture

Primary rat NP cells were obtained from Procell Life Science & Technology (CP-R145, Wuhan, China), which were extracted from the intervertebral disc tissue of SD rats. NP cells were cultured in complete medium supplemented with 10% fetal bovine serum (Gibco, Grand Island, NY, United States) and 1% penicillin/streptomycin (Gibco) in a 5% CO2 incubator at 37° C condition. Nucleus pulposus cells were cultured in a 24-well plate (5×104/well) and incubated at 37°C for 24 h. The various treatments were applied to the cells for 24 h. The S group was treated with PBS. The M group was stimulated by adding the proinflammatory factors IL-1β (50 ng/mL) (25, 26).

### 2.11 Western blotting analysis

Total protein was extracted from rat NP cells with the radio immunoprecipitation assay (RIPA) buffer containing phosphatase and protease inhibitors. Then, bicinchoninic acid (BCA) protein assay kit was used to determine the protein concentrations. Thirty micrograms of total protein were separated by sodium dodecyl sulfate polyacrylamide gel electrophoresis (SDS-PAGE), and then electro-transferred to the transmembrane PVDF (Millipore, Billerica, MA, USA), blocked with 5% milk protein for 1 h. After that, the target membranes were incubated with following primary antibodies overnight at 4°C: MMP9 antibody (1:1000, Proteintech, Wuhan, China), UCHL1antibody (1:1000, Proteintech, Wuhan, China), HPGD antibody (1:2000, Proteintech,Wuhan,China), GAPDH (1:2000, Antgene, Wuhan, China). Following incubation with the appropriate secondary antibody (goat anti-rabbit or goat anti-mouse; KPL, USA), the membrane was developed using a chemiluminescent substrate. The resulting bands were visualized and subjected to densitometric analysis with Quantity One software (Bio-Rad, USA).

### 2.12 Statistical analysis

All statistic data were expressed as mean ± SD, and firstly evaluated for normal distribution using Shapiro-Wilk test. Then, the results were compared by one-way ANOVA followed with Tukey’s or Games-Howell’s *post hoc* test. The Kruskal-Wallisnon-parametric test was also used to evaluate non-normal distribution variables. The value of P<0.05 was considered statistically significant. The Graph Pad Prism 5.0 software (San Diego, CA, USA) was employed to perform the statistical analysis. The datasets supporting this study’s findings are fully provided within the manuscript and its supplemental materials.

## 3 Results

### 3.1 Data integration and identification of DEGs

**Figure 1:**
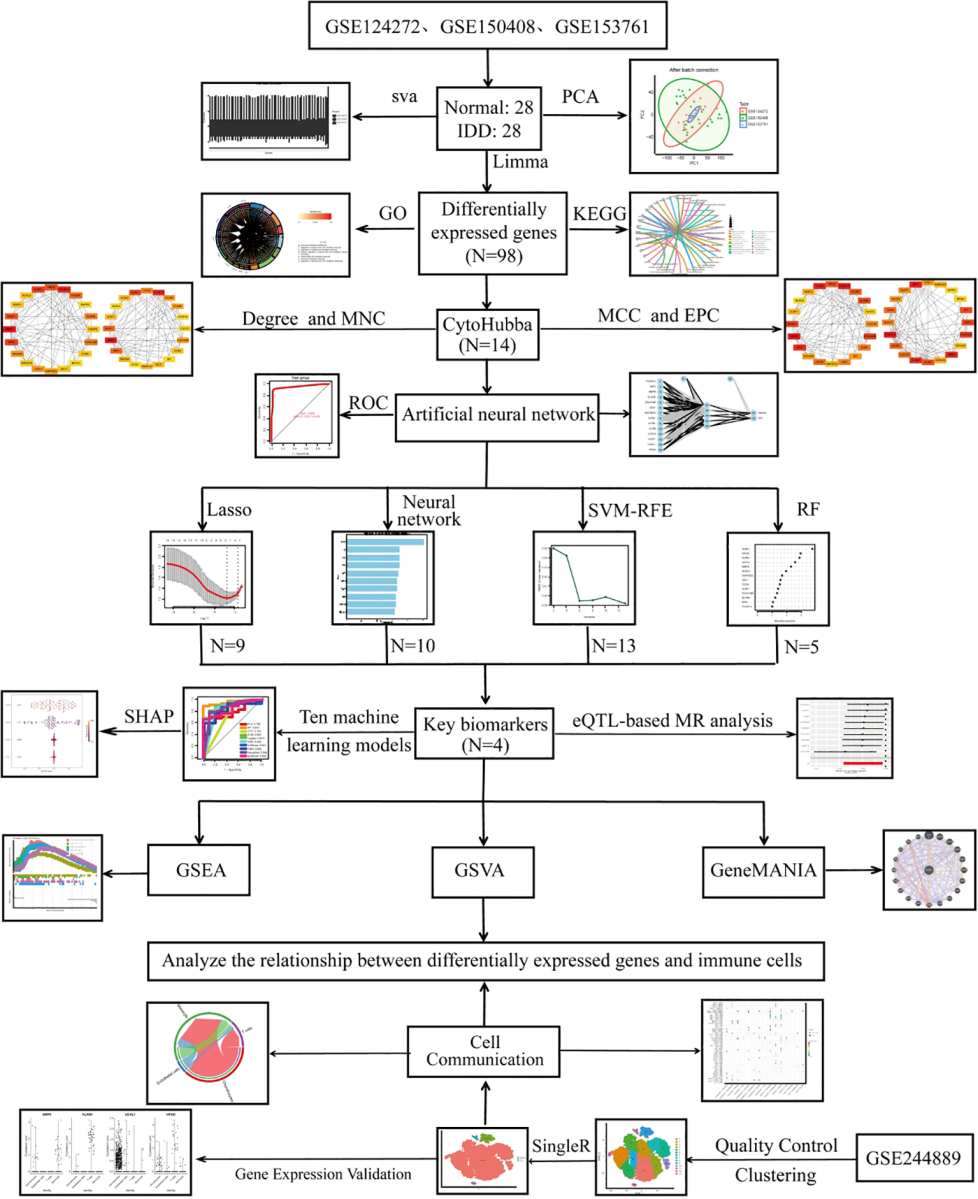
Flowchart of this study.

**Figure 2:**
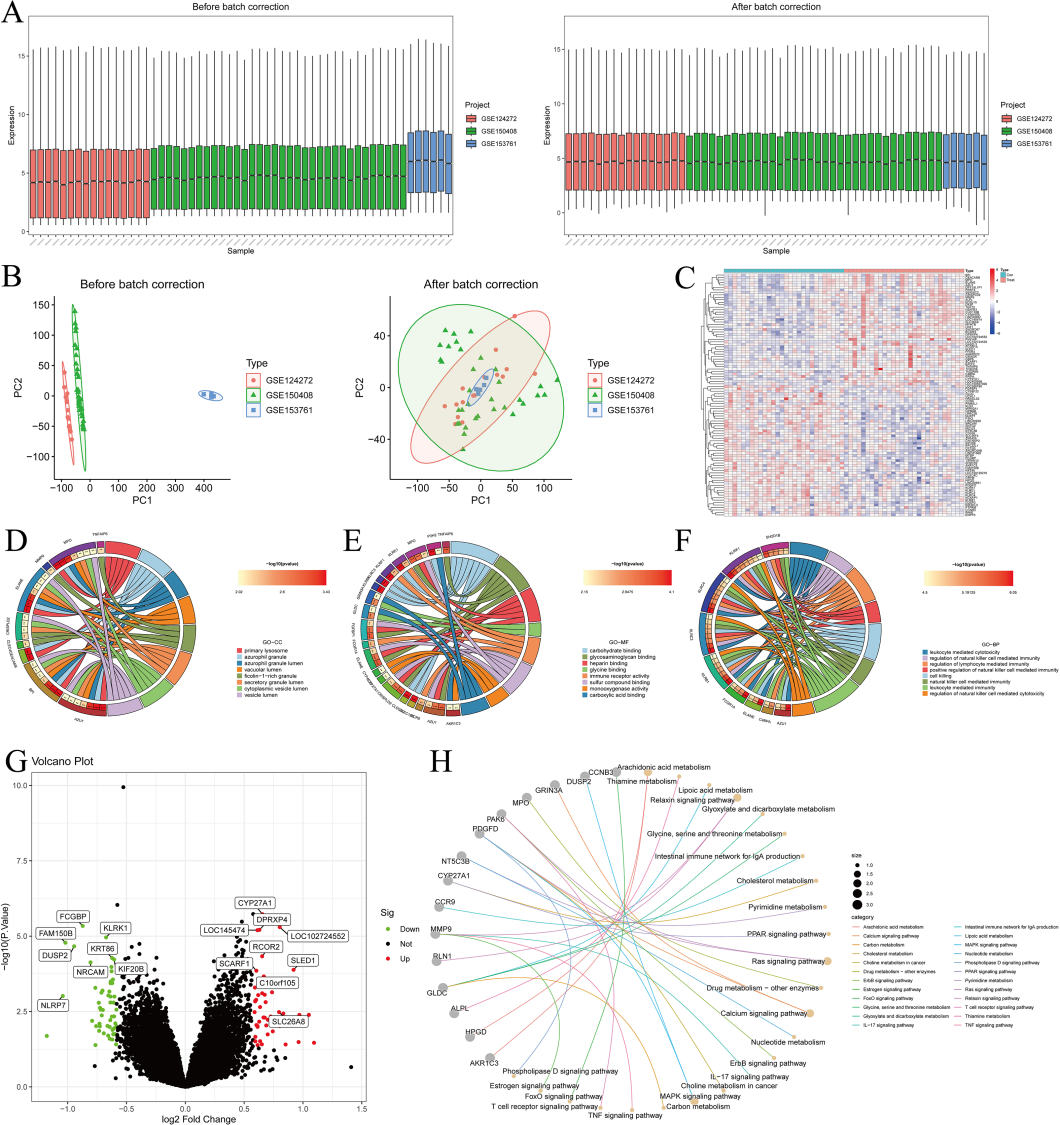
Differential expression analysis, batch effect correction, and functional enrichment analysis. **(A, B)** PCA analysis showing samples from different experiments before batch correction, with random shuffling; **(C)** Heatmap of DEGs in the IDD dataset; **(D–F)** GO enrichment analysis, including BP, CC, and MF; **(G)** Volcano plot of DEGs in the IDD dataset; **(H)** KEGG pathway enrichment analysis.

**Figure 1** illustrates the overall workflow of this study. Before batch correction, samples from different experiments were clearly separated, indicating a pronounced batch effect. After applying PCA, the samples from different experiments appeared randomly distributed, suggesting that the batch effect was effectively mitigated (**Figures 2A, B**). By applying the Limma algorithm, we identified a total of 98 differentially expressed genes (DEGs), among which 45 were significantly upregulated and 53 were downregulated (**Figures 2C, G**).

### 3.2 Functional enrichment analysis

The GO enrichment analysis was categorized into three domains: biological processes (BP), cellular components (CC), and molecular functions (MF). BP results showed enrichment in leukocyte- and lymphocyte-mediated immunity, cytotoxicity, and defense response to bacteria (**Figure 2D**). CC analysis indicated that genes were mainly located in secretory granules, lysosomes, and vesicle lumens, suggesting roles in vesicle transport and immune-related granule function (**Figure 2E**). MF analysis showed enrichment in oxidoreductase activity, phosphatase activity, and various binding functions (e.g., organic acids, glycosaminoglycans, and carbohydrates), indicating involvement in metabolic regulation, immune response, and extracellular matrix interactions (**Figure 2F**).

KEGG analysis showed enrichment in IL-17, TNF, PPAR, MAPK, and T cell receptor signaling pathways, which are closely related to inflammation and immune regulation in IDD. Pathways such as FoxO, Ras, calcium, relaxin, and ErbB signaling further suggested potential roles in cell survival, extracellular matrix homeostasis, and stress responses. Moreover, enrichment in cholesterol and arachidonic acid metabolism indicates that metabolic dysregulation may contribute to disc degeneration and inflammatory processes (**Figure 2H**).

### 3.3 Identification of hub genes via PPI network analysis

**Figure 3:**
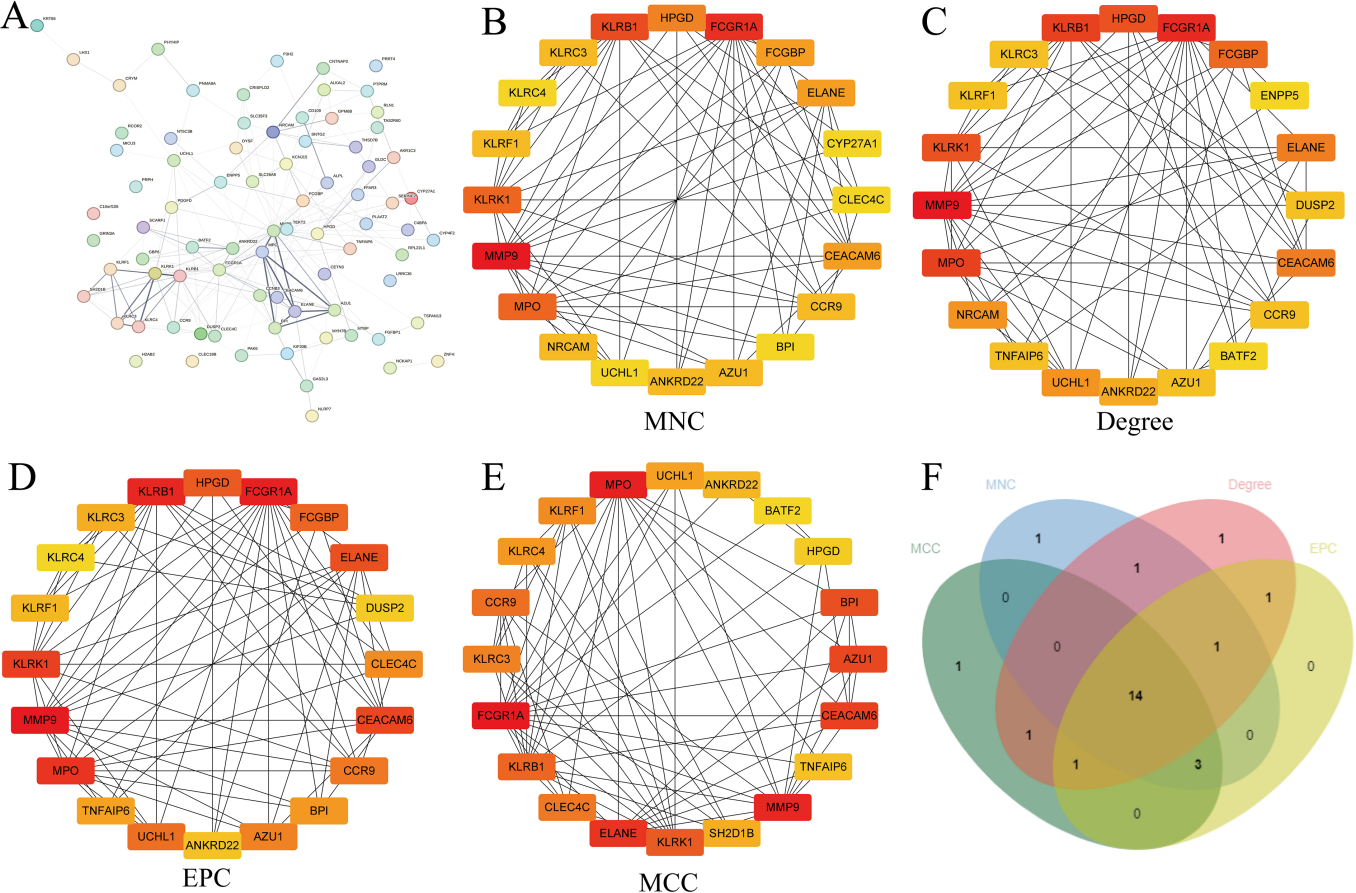
Identification of hub genes using CytoHubba. **(A)** STRING network visualization of DEGs; Hub genes identified using different CytoHubba scoring methods: MNC **(B)**, Degree **(C)**, EPC **(D)**, and MCC **(E, F)** Venn diagram showing overlapping hub genes identified by different methods.

The 98 DEGs were visualized using the STRING database (**Figure 3A**). To prioritize potential therapeutic targets, these DEGs were analyzed with four topological algorithms—MCC, EPC, Degree, and MNC—to identify hub genes (**Figures 3B–E**). The intersection of the top 20 genes from each method revealed 14 hub genes (**Figure 3F**).

### 3.4 ANN prediction results

**Figure 4:**
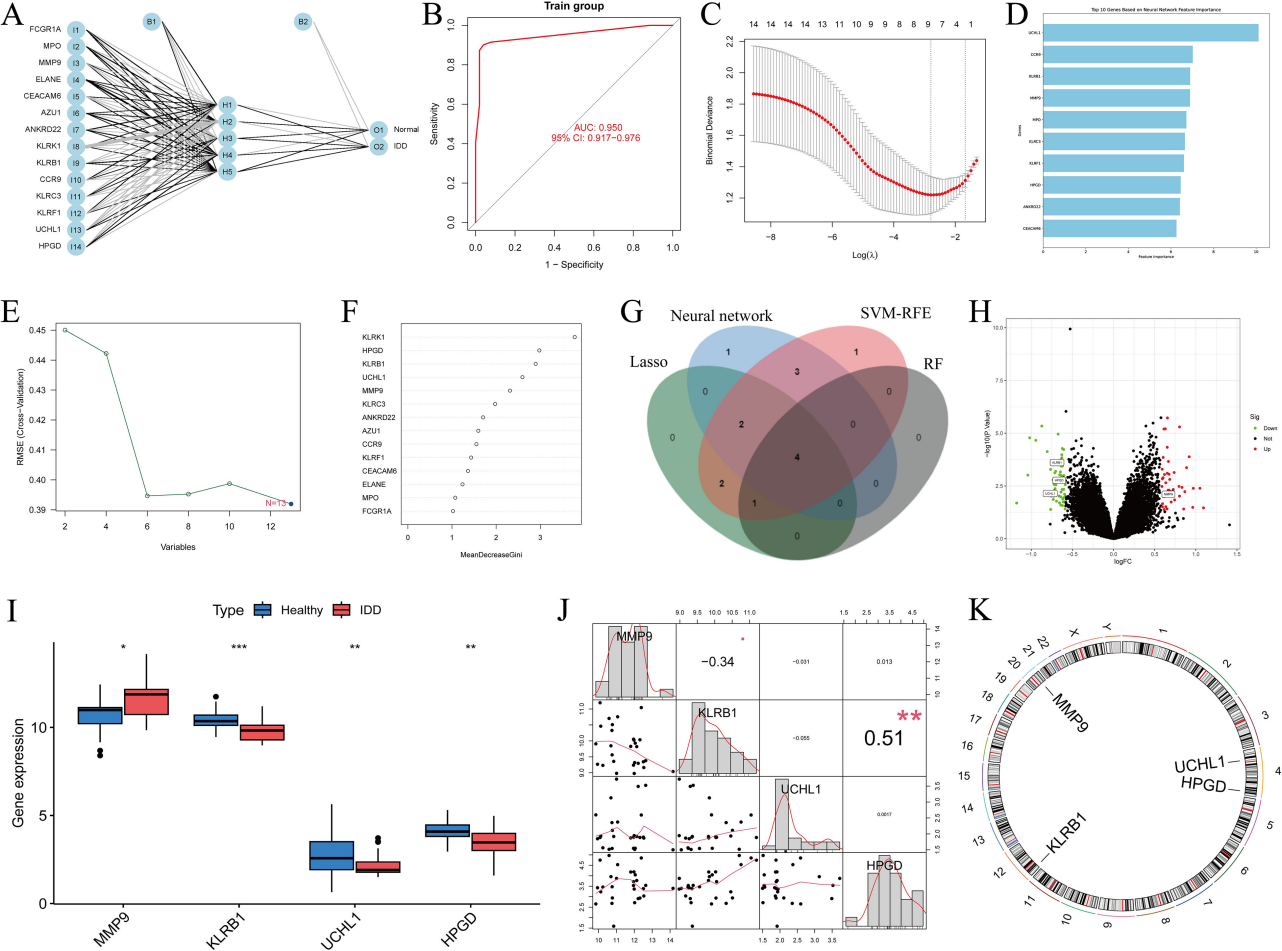
Screening of DEGs using multiple methods and visualization of the results. **(A, B)** ANN distinguishing the experimental group from the control group, with ROC curves constructed to evaluate overall diagnostic performance; **(C)** Gene selection using the LASSO algorithm; **(D)** Visualization of the top 10 important genes using the neural network algorithm; **(E)** Gene selection using the SVM-RFE algorithm; **(F)** Gene selection using the random forest algorithm; **(G)** Intersection of the results from the four algorithms, yielding four genes; **(H)** Volcano plot of DEGs; **(I)** Boxplot of DEGs; **(J)** Relationships among the four genes; **(K)** Chromosomal locations of the intersecting genes labeled on the corresponding chromosomes. *: *p* < 0.05, **: *p* < 0.01, ***: *p* < 0.001.

The artificial neural network (ANN) constructed in this study consisted of an input layer, a hidden layer with five nodes, and an output layer. The model was trained using the Rprop optimization algorithm and the SSE loss function, and its classification performance was evaluated with a confusion matrix. Our results showed that the ANN achieved an accuracy of 93.6% in the experimental group and 96.4% in the control group, with an overall prediction accuracy of 95% (**Figures 4A, B**).

### 3.5 Feature gene selection based on machine learning

We applied LASSO regression to generate cross-validation curves and identified nine disease-characteristic genes (**Figure 4C**). Using the neural network algorithm, the top 10 characteristic genes ranked by importance were selected (**Figure 4D**). The SVM-RFE method produced accuracy and cross-validation error curves, from which 13 characteristic genes were identified (**Figure 4E**). Using the Random Forest (RF) algorithm, we generated a decision tree scoring plot and ranked gene importance, from which five key genes were identified (**Figure 4F**). Subsequently, a Venn diagram illustrated the overlap among genes selected by all four machine learning approaches, revealing four intersecting candidates (**Figure 4G**; **Supplementary Table S2**).

Additionally, we constructed box plots of the DEGs and combined them with a volcano plot, which revealed that MMP9 was upregulated in the experimental group, whereas KLRB1, UCHL1, and HPGD were downregulated. Furthermore, the correlations among these four genes and their chromosomal distributions were visualized using a circos plot (**Figures 4H–K**).

### 3.6 SHAP analysis results

**Figure 5:**
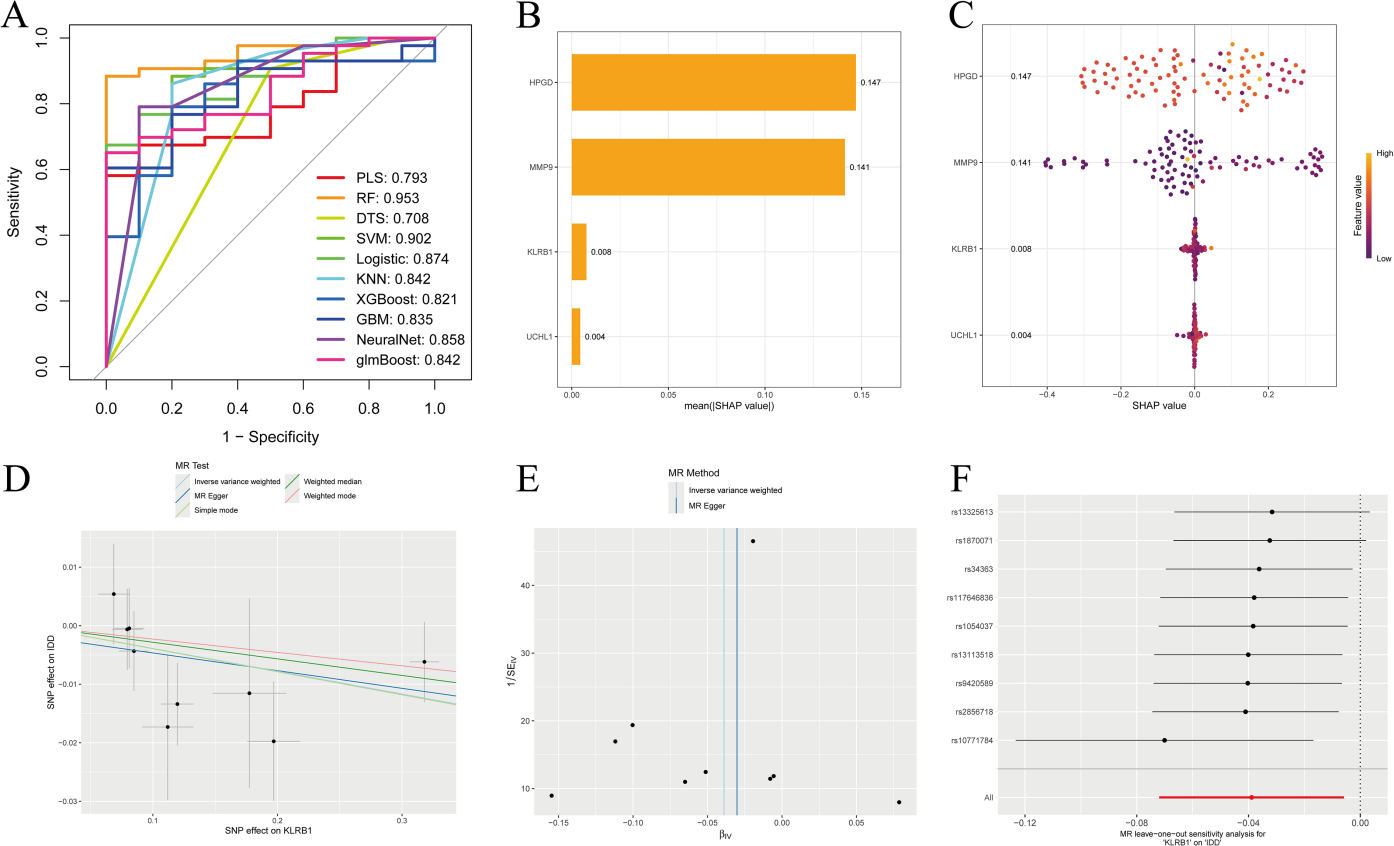
SHAP analysis and eQTL-based MR analysis. **(A)** ROC curves constructed using ten machine learning models to evaluate overall diagnostic performance; **(B)** Bar chart with the vertical axis representing gene names and the horizontal axis representing the mean absolute SHAP value. Higher values indicate a greater impact of the gene on the prediction results; **(C)** Bee swarm plot with the vertical axis representing gene names and the horizontal axis representing SHAP values, used to calculate the mean SHAP value for each gene; **(D–F)** MR analysis of the KLRB1 gene, including scatter plot, funnel plot, and leave-one-out analysis plot.

We evaluated the diagnostic performance of four IDD DEGs using ten machine learning models combined with 10-fold cross-validation, and assessed overall performance with ROC curves (**Figure 5A**). For predictive performance analysis, bar charts and bee swarm plots were generated: in the bar chart, higher values indicate greater impact of a gene on the prediction; the bee swarm plot shows the mean SHAP value of each gene, reflecting its contribution to the model (**Figures 5B, C**). Through SHAP analysis, we not only interpreted the machine learning model but also quantified the importance of each gene. The results demonstrated that gene expression could effectively distinguish patients at the single-sample level. By integrating these findings, we achieved a comprehensive explanation of the model’s interpretability.

### 3.7 Genetically causal target identification through MR analysis

MR analysis using expression quantitative trait loci (eQTL) was conducted to evaluate genetic causality. Based on the Inverse Variance Weighted (IVW) approach, KLRB1 was the only one among the four candidate genes to show a significant association with IDD, suggesting a potential protective role (**Figures 5D–F**). The other three genes were not identified, which may be due to the dataset containing data exclusively from European populations.

### 3.8 Functional enrichment analysis of core genes

**Figure 6:**
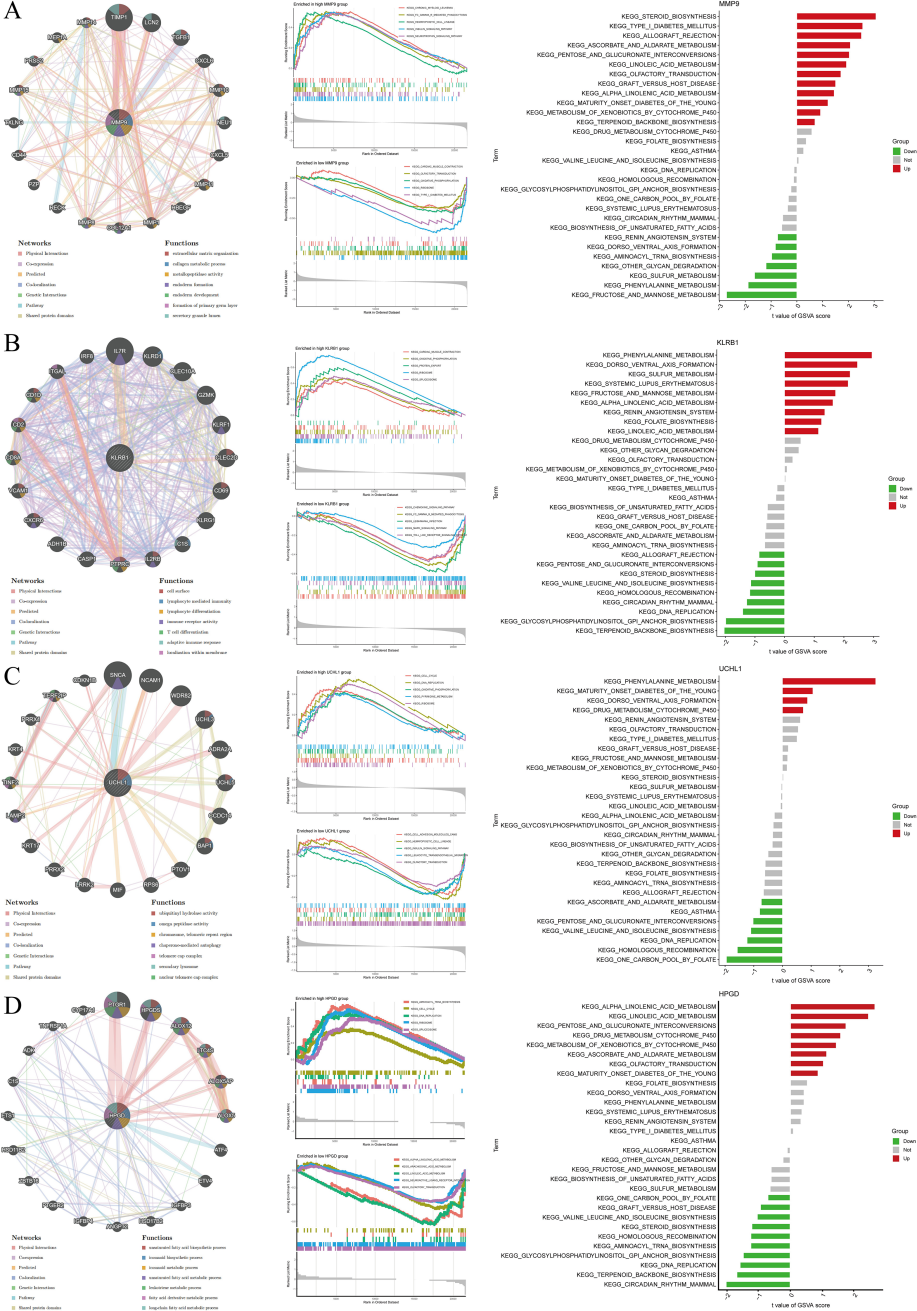
Visualization of GeneMANIA, GSEA, and GSVA results for target gene analysis. **(A)** MMP9; **(B)** KLRB1; **(C)** UCHL1; **(D)** HPGD.

GeneMANIA, GSEA, and GSVA were employed to investigate functional and pathway enrichment between the high- and low-expression groups of the target genes, and the top enriched pathways were visualized. MMP9 high expression may promote intervertebral disc degeneration by driving extracellular matrix degradation and shaping an immune-inflammatory microenvironment, whereas low expression may contribute to the maintenance of energy metabolic homeostasis (**Figure 6A**). KLRB1 may play a pivotal role in IDD by regulating energy metabolism and immune-inflammatory responses, acting as an important immune-regulatory molecule on the cell membrane involved in disease progression (**Figure 6B**). UCHL1 may influence IDD progression by modulating cell proliferation, metabolism, DNA repair, and immune responses (**Figure 6C**). HPGD may contribute to IDD through regulation of cell proliferation, gene expression, lipid metabolism, and inflammatory mediator signaling (**Figure 6D**). Overall, these genes collectively mediate metabolism, immunity, and matrix remodeling in IDD.

### 3.9 Immune cell infiltration

**Figure 7:**
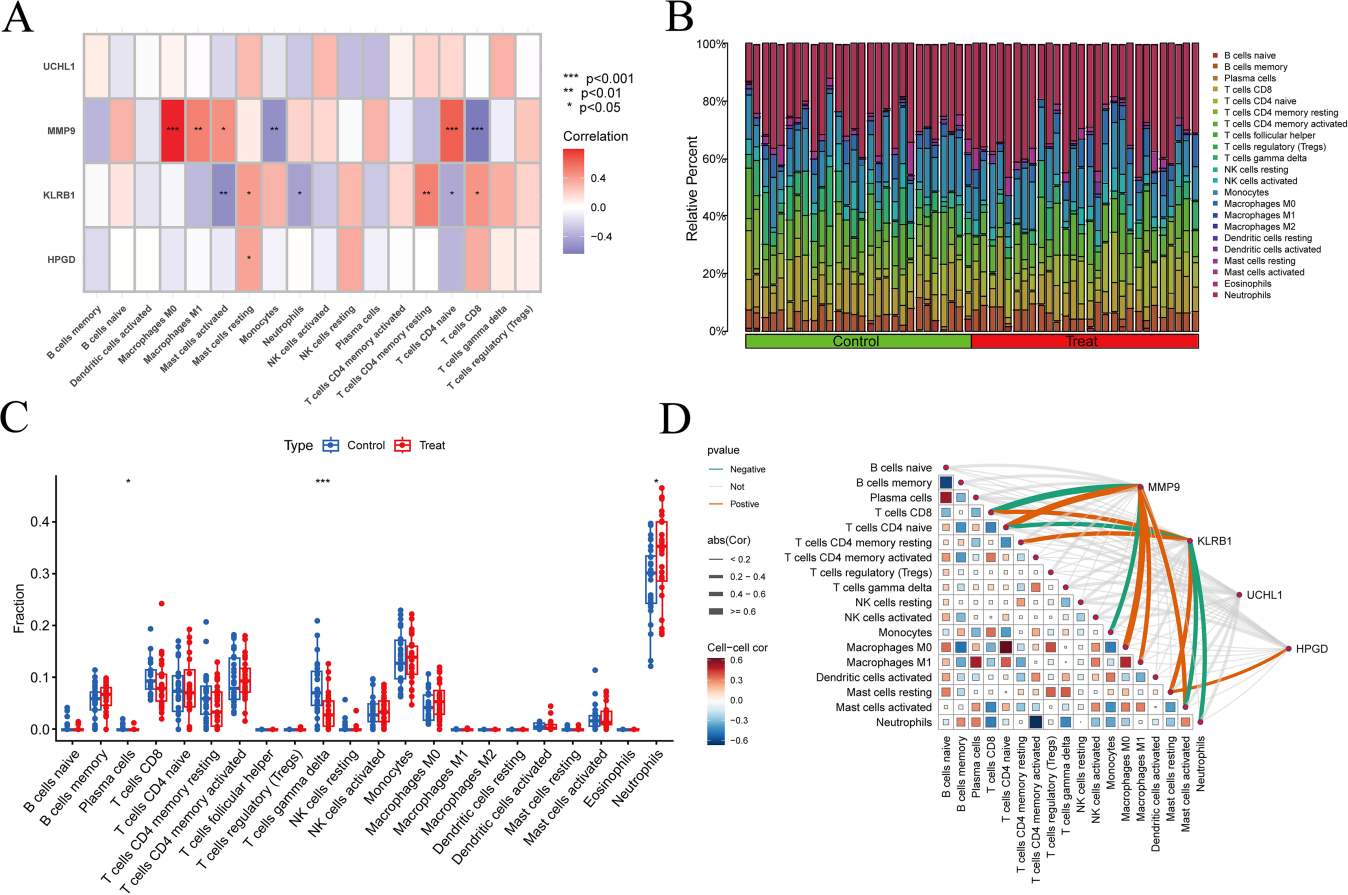
Immune-related analysis results. **(A, D)** Relationships between immune cells and target genes; **(B)** The number of immune cells in each sample was determined through immune cell infiltration analysis. The results were visualized as a bar chart, with the horizontal axis representing samples and the vertical axis representing immune cell content. The sum of all immune cell contents equals 1. Different colors indicate different types of immune cells; **(C)** Box plot of differences, with the horizontal axis representing immune cell types and the vertical axis representing immune cell content. Green represents the control group, and red represents the experimental group, * *p* < 0.05, ** *p* < 0.01, *** *p* < 0.001.

**Figure 8:**
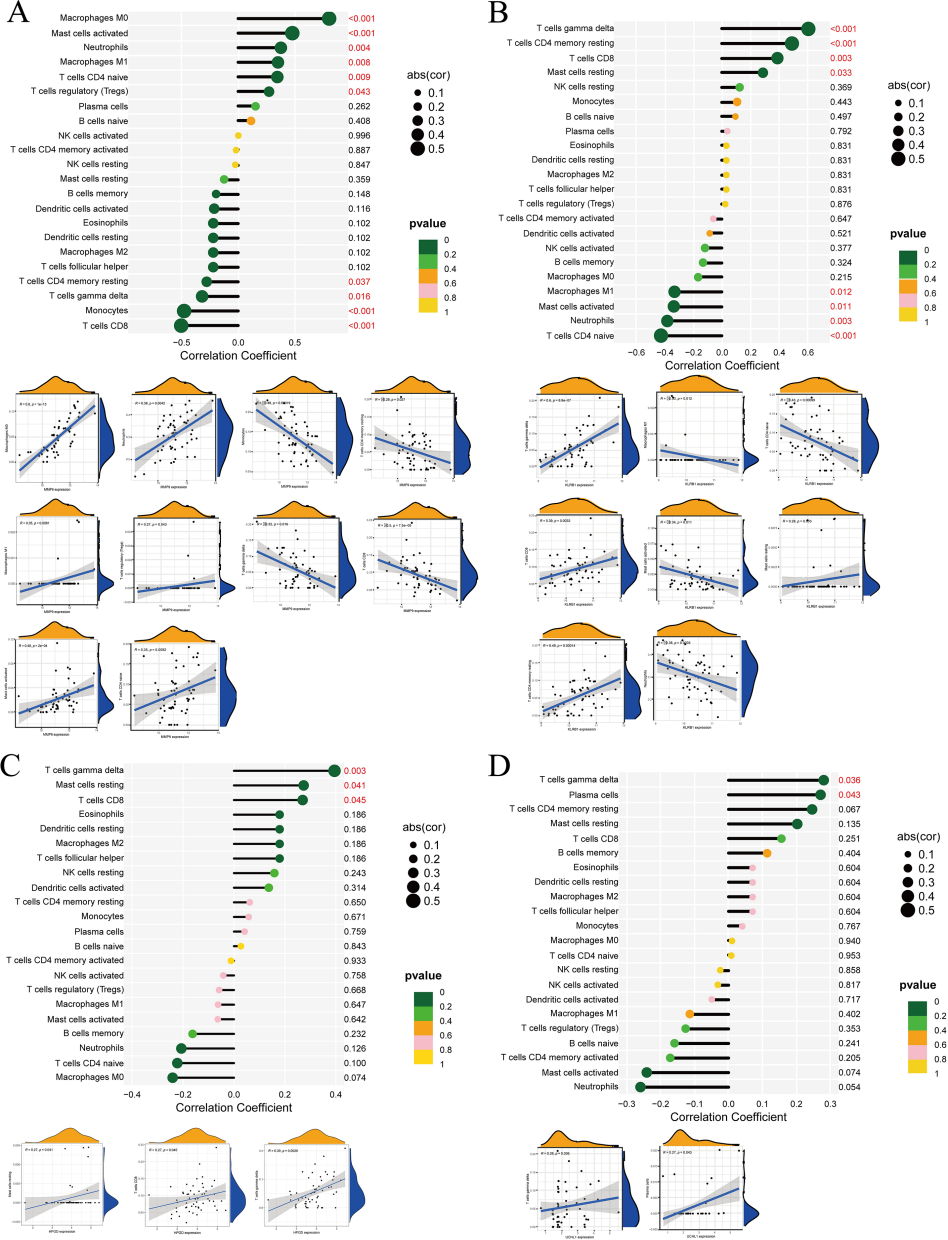
Correlation analysis between DEGs and immune cells. **(A)** MMP9, **(B)** KLRB1, **(C)** HPGD, **(D)** UCHL1. In the correlation bubble plots, the vertical axis represents immune cell types, the horizontal axis represents the correlation coefficient, the size of the circle represents the absolute value of the correlation coefficient, and the color of the circle represents the P value of the correlation test. In the scatter plots, the horizontal axis represents the expression level of the target gene, the vertical axis represents the content of immune cells, R represents the correlation coefficient, and P represents statistical significance.

In the differential box plots, an asterisk above an immune cell denotes a statistically significant difference between the control and experimental groups for that cell type (**Figures 7A–C**). We further analyzed the relationships between immune cells and MMP9, KLRB1, HPGD, and UCHL1, finding that UCHL1 showed a relatively weak overall association with immune cells (**Figure 7D**). In addition, all four genes were related to T cells gamma delta, but the expression of MMP9 exhibited an opposite trend with T cells gamma delta counts compared to the other three genes. The correlation results were then visualized using lollipop plots (**Figures 8A–D**) to clearly illustrate the interactions between these genes and immune cells.

### 3.10 Single-cell transcriptome analysis

Single-cell RNA sequencing data from three IDD patients were first quality-filtered to remove low-quality cells. Gene expression patterns across the retained cells were visualized using feature plots (**Supplementary Figure S1A**), and 1,500 highly variable genes were selected for downstream analysis (**Supplementary Figure S1B**). Principal component analysis (PCA) revealed clear segregation of individual samples, indicating minimal batch effects (**Supplementary Figure S1C**). Clustering via t-SNE identified 14 distinct cell populations, which were subsequently classified into four major immune cell types based on canonical markers and annotations from the SingleR package (**Supplementary Figures S1D, E**). Analysis of core target gene expression across these subpopulations showed that MMP9 was primarily expressed in monocytes, KLRB1 in T cells, and UCHL1 predominantly in chondrocytes, highlighting their potential cell type-specific contributions to IDD pathogenesis (**Supplementary Figure S1F**).

### 3.11 Cell communication mapping in IDD microenvironment

**Figure 9:**
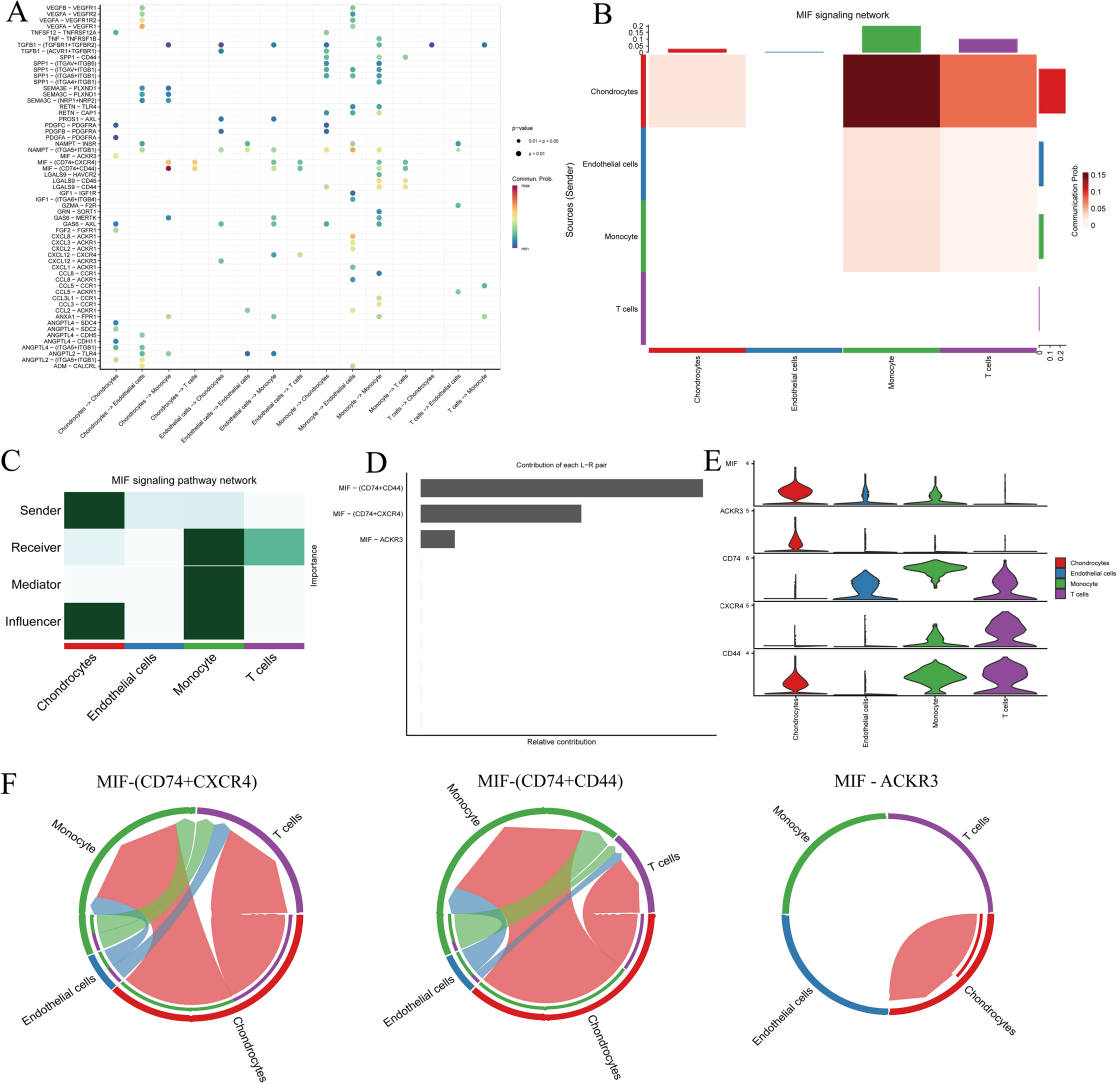
Cell–cell communication analysis of IDD cell subsets with distinct differentiation patterns. **(A)** Dot plot of ligand–receptor (L–R) pairs in subtype-specific pathways; **(B, C)** Network centrality scoring analysis; **(D)** Contribution of individual receptors to the overall MIF signaling pathway; **(E)** Expression distribution of genes associated with the MIF pathway; **(F)** Co-expression of CD74^+^CXCR4 and CD74^+^CD44 across different cell types.

In single-cell ligand–receptor signaling enrichment analysis based on 98 DEGs in IDD, the MIF pathway was significantly enriched, with chondrocytes serving as the primary signal sources and monocytes and T cells as the main recipients (**Figures 9A–C**). Signal transduction was largely mediated via the CD74–CD44 and CD74–CXCR4 ligand–receptor pairs (**Figures 9D, E**). Notably, although chondrocytes transmit signals to monocytes and T cells, these interactions appear absent in endothelial cells, suggesting cell type-specific signaling dynamics within the IDD microenvironment (**Figure 9F**).

### 3.12 Experimental results

**Figure 10:**
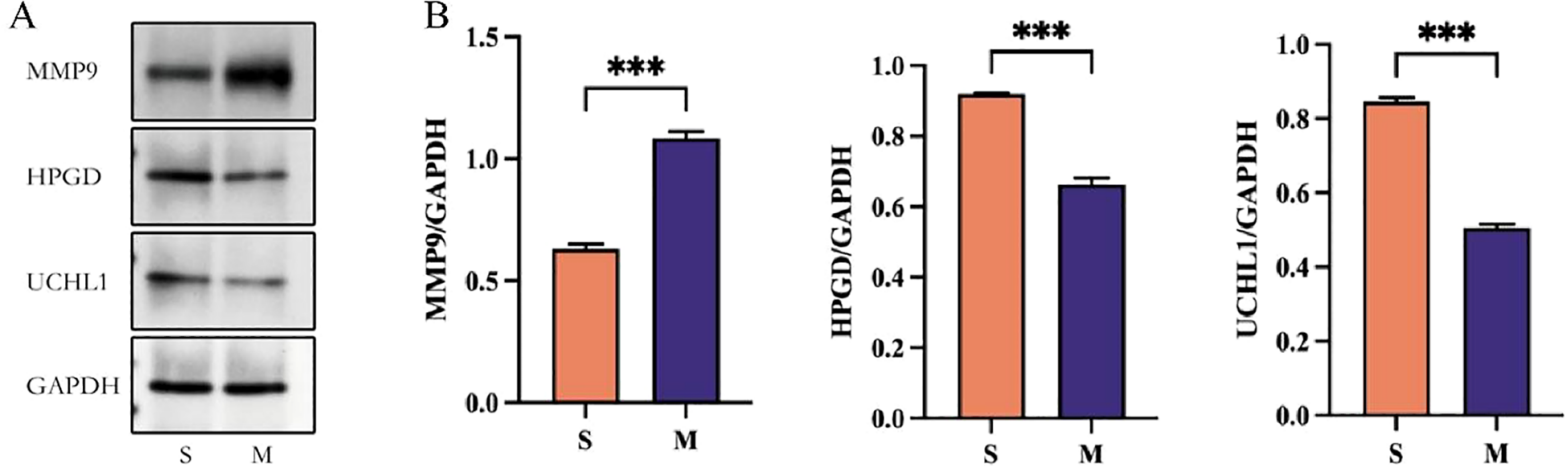
Experimental validation. Western blot were used to detect the expression level of MMP9, HPGD, and UCHL1 in S group and M group.

Primary rat nucleus pulposus (NP) cells were used as the research model. In the experimental design, the S group was treated with PBS, while the M group (IDD) was stimulated with 50 ng/mL IL-1β in the culture medium to establish an inflammatory model for 24 h. To investigate the regulatory roles of UCHL1, HPGD, and MMP9 in intervertebral disc degeneration, Western blotting was employed. As demonstrated in **Figures 10A, B**, compared with the S group, the expression level of MMP9 was significantly increased (P<0.05), while the expression levels of UCHL1 and HPGD were markedly decreased in the M group (P<0.05). This suggested that inflammatory stimulation may cause protein expression imbalance in NP cells and play an important role in the pathological process.

## 4 Discussion

IDD is a major contributing factor (27, 28). High-throughput sequencing combined with bioinformatics can help identify key biomarkers of IDD and provide new avenues for treatment (29, 30). In this study, we conducted a comprehensive gene-level analysis to systematically elucidate the differences in gene expression, signaling pathway activity, and immune responses in IDD. We integrated PPI networks, ANN, multiple ML approaches, SHAP interpretation analysis, MR, and single-cell DEGs analysis to explore the contribution of DEGs to IDD diagnosis. Furthermore, we investigated the changes of these genes in immune responses and examined the interactions and correlations among immune cells, providing insights into the molecular mechanisms of IDD from both genetic and immune perspectives. Finally, single-cell analysis of cell–cell communication revealed key ligand–receptor interactions and signaling pathways between chondrocytes and immune cells.

We evaluated the potential of KLRB1, MMP9, UCHL1, and HPGD as diagnostic biomarkers using Western blot assays. Further analysis indicated that KLRB1 showed no significant difference in expression between IDD patients and healthy individuals, suggesting its limited value as a potential diagnostic marker. Our analysis revealed that the selected genes are closely associated with immune function, and their upregulation in the inflammatory IDD group may therefore have biological significance. Moreover, MMP9, UCHL1, and HPGD exhibited strong discriminatory ability between healthy individuals and IDD patients, representing a novel finding of this study. Western blot validation further confirmed that MMP9, UCHL1, and HPGD consistently maintained significant discriminatory power.

In this study, we performed functional enrichment analysis of DEGs associated with IDD, revealing several key biological processes and signaling pathways, including TNF, IL-17, PPAR, MAPK, and FoxO pathways. TNF-α is abnormally expressed in degenerated discs and contributes to inflammation and ECM degradation (31). IL-17 expression correlates positively with IDD severity, regulating ECM metabolism, inflammation, angiogenesis, and NP cell autophagy and proliferation (32). PPAR plays a protective role in IDD, and its agonists can alleviate IL-17-induced inflammation and degeneration by inhibiting NF-κB signaling (33). The p38 MAPK pathway is involved in inflammation, ECM degradation, apoptosis, and stress responses, representing a potential therapeutic target (34). FoxO transcription factors are critical for maintaining disc homeostasis, and their loss can promote degeneration (35). Together, these findings suggest that IDD is a multifactorial disease involving complex interactions among multiple signaling pathways. Future studies should further elucidate the crosstalk and mechanisms of these pathways to identify novel therapeutic targets for IDD.

In this study, we successfully identified four potential biomarkers for IDD diagnosis, namely MMP9, KLRB1, HPGD, and UCHL1, which play important roles in immune function. By constructing ANN and evaluating its diagnostic performance, ROC curve analysis indicated that the ANN achieved a prediction accuracy of 95%. Subsequently, we developed multiple machine learning models using these biomarkers and performed SHAP analysis to provide interpretability, revealing the contribution and potential mechanisms of each gene in IDD diagnosis. Furthermore, MR analysis based on gene eQTL data suggested a potential causal relationship between KLRB1 and IDD, indicating a possible protective effect. However, other candidate genes did not show significant causal associations, which may be attributable to limitations such as small sample size, low gene expression levels, or insufficient gene–phenotype association signals. Future studies with larger multi-omics datasets and independent cohorts are needed to further elucidate the roles of these genes in IDD pathogenesis.

Studies have shown that matrix metalloproteinase 9 (MMP-9) plays a key role in the onset and progression of IDD. MMP-9 degrades extracellular matrix components in the disc, such as collagen and proteoglycans, and its expression level is positively correlated with the severity of disc degeneration and herniation (36). In addition, the -1562C/T polymorphism of the MMP-9 gene is associated with IDD susceptibility in young adults (37), and downregulation of miR-133a promotes collagen II loss by targeting MMP-9, further contributing to disc degeneration (38). These findings suggest that MMP-9 may serve as a potential biomarker and therapeutic target for IDD.

UCHL1 (Ubiquitin C-terminal hydrolase L1) is closely associated with IDD. Transcriptomic analyses and clinical sample validation have demonstrated that UCHL1 expression is significantly downregulated in both peripheral blood and intervertebral disc tissues of IDD patients, suggesting its potential involvement in the pathogenesis of disc degeneration (39). Further mechanistic studies have revealed that UCHL1 regulates HSPA8 through deubiquitination, thereby activating the chaperone-mediated autophagy pathway, which plays a critical protective role in delaying intervertebral disc degeneration (40).

In the pathological context of IDD, previous studies have shown that immune cells such as T cells and NK cells infiltrate the degenerative disc tissue. By releasing pro-inflammatory cytokines including IL-1β, TNF-α, and IL-6, they exacerbate cell apoptosis and ECM degradation, thereby promoting IDD progression (22). The protein level of HPGD is significantly increased during the symptomatic and late stages of motor neuron disease (41). These results suggest that HPGD may participate in inflammatory responses and pathological processes in the nervous system by regulating the metabolism of prostaglandin E2.

Our study revealed significant differences in plasma cells, γδ T cells, and neutrophils between the control and experimental groups. We then further explored the relationships between individual genes and immune cells, and the results showed that UCHL1 exhibited relatively weak associations with immune cells. In a more detailed analysis of gene–immune cell interactions, we found that MMP9 displayed significant differences in the following immune cell types: M0 macrophages, CD8 T cells, monocytes, activated mast cells, neutrophils, M1 macrophages, naïve CD4 T cells, γδ T cells, resting memory CD4 T cells, and regulatory T cells (Tregs). At the same time, KLRB1 showed significant differences in γδ T cells, resting memory CD4 T cells, naïve CD4 T cells, CD8 T cells, neutrophils, activated mast cells, M1 macrophages, and resting mast cells. Further analysis demonstrated that HPGD was more strongly associated with γδ T cells, CD8 T cells and mast cells resting. For UCHL1, significant differences were observed in plasma cells and γδ T cells. Notably, when all four DEGs were altered, γδ T cells consistently exhibited differential changes. However, the expression pattern of MMP9 in immune cells was not entirely consistent with that of the other three core genes.

In this study, we found that γδ T cells may play a potential role in intervertebral disc (IVD) injury and degeneration. Clayton et al. reported that γδ T cells predominantly appear in injured female IVD tissues and exert anti-inflammatory and tissue-protective effects, which may explain the sex differences in disc degeneration; male mice, lacking the protection of γδ T cells, showed more severe degeneration (42). Interestingly, in studies on bone fracture repair, mice deficient in γδ T cells exhibited more mature tissue regeneration, stronger expression of bone and cartilage matrix proteins, and improved biomechanical strength, suggesting that γδ T cells may delay bone healing in certain contexts (43). These findings not only deepen our understanding of the immune mechanisms underlying IDD but also highlight the need for future research to explore the spatiotemporal roles of γδ T cells in tissue injury and repair, as well as their potential therapeutic value.

CD4^+^ T cells also play an essential role in the adaptive immune system and are closely involved in the pathogenesis of IDD. Several studies have demonstrated that the proportion of resting memory CD4^+^ T cells is significantly decreased in degenerated disc tissues, suggesting impaired immune homeostasis in IDD patients (39). In contrast, specific subsets such as Th17 cells are enriched in degenerative discs and can secrete pro-inflammatory cytokines including IL-17 and TNF-α, thereby accelerating extracellular matrix degradation and promoting disease progression (8, 22). On the other hand, Treg cells exert immunosuppressive effects through IL-10 and TGF-β, and their functional imbalance with Th17 cells is considered a hallmark of IDD-associated immune dysregulation (44). Furthermore, a recent Mendelian randomization analysis indicated a bidirectional causal relationship between CD39^+^ CD4^+^ T cells and IVDD, highlighting their potential as both biomarkers and therapeutic targets (45). Collectively, these findings indicate that CD4^+^ T cells, through their diverse subsets and cytokine profiles, exert both pathogenic and protective functions in IDD, and the imbalance between them may critically shape disease onset and progression.

Through single-cell analysis of nucleus pulposus tissue, we found that chondrocytes can regulate the function of monocytes via the MIF signaling pathway, thereby participating in the modulation of the intervertebral disc immune microenvironment and the degenerative process. Previous studies have shown that MIF expression is upregulated in degenerated nucleus pulposus cells and promotes extracellular matrix degradation and apoptosis via the NF-κB signaling pathway, and the use of the MIF inhibitor CPSI-1306 can alleviate disc degeneration in mouse models, suggesting that MIF may serve as a potential therapeutic target for IDD (46). Furthermore, single-cell RNA sequencing studies revealed that MIF can regulate nucleus pulposus cell function and extracellular matrix metabolism through binding to the ACKR3 receptor, with MIF expression positively correlated with ACKR3, and MIF inhibition mitigating degenerative changes in nucleus pulposus cells (47). These studies further support the potential of MIF inhibitors in alleviating disc degeneration, reducing nucleus pulposus cell apoptosis, and suppressing inflammation (48). Therefore, our single-cell analysis findings are consistent with existing literature, indicating that MIF may play a central role in chondrocyte–immune cell interactions and providing new insights for immune-based therapeutic strategies for IDD.

In this study, we employed a multi-level analytical strategy to explore the molecular mechanisms of IDD. First, PPI networks were constructed to identify key gene nodes. Then, ANNs and machine learning were used to evaluate the predictive value of these genes, with SHAP analysis quantifying each gene’s contribution to the models. Subsequently, MR was applied to validate the causal relationships between key genes and the disease. Finally, single-cell sequencing data were integrated to analyze the expression patterns of differentially expressed genes in specific immune cell types. This approach systematically reveals core genes and immune features in IDD, providing a reference for future precision diagnosis and intervention. However, the following limitations should be considered when interpreting our results. First, this study did not perform microarray or RNA sequencing, and the gene expression data were entirely obtained from the GEO database. In addition, the IDD cell model used in this study could not fully recapitulate the complex *in vivo* IDD microenvironment, which is far more physiologically intricate than *in vitro* models. Finally, the GWAS dataset used in this study was primarily derived from European populations, which may limit the applicability of the findings to other populations. Future studies could include cohorts from diverse ethnic groups to enhance the generalizability and representativeness of the results. Moreover, the identified key genes and pathways may serve as potential biomarkers or therapeutic targets, providing new insights for early diagnosis and individualized treatment of IDD.

## 5 Conclusion

In this study, IDD was analyzed by integrating ANN, multiple machine learning methods, SHAP analysis, and MR. During gene feature selection, we applied ANN, LASSO, SVM-RFE, and RF, and subsequently validated the results using Western blot experiments. Ultimately, three potential biomarkers were identified: MMP9, HPGD, and UCHL1.By combining ten machine learning approaches with SHAP models, we systematically evaluated the role of these core genes in IDD diagnosis, revealing the genetic characteristics, molecular pathways, and differentially abundant immune cell types associated with IDD. Further integration with single-cell analysis allowed us to explore the potential role of the MIF pathway in IDD pathogenesis. Overall, our findings indicate that machine learning–based approaches can provide effective support for the precise diagnosis of IDD, while also offering novel insights for the development of clinical interventions and therapeutic strategies.

## Data Availability

The datasets presented in this study can be found in online repositories. The names of the repository/repositories and accession number(s) can be found in the article/**Supplementary Material**.
