# Supplementary material for: Disc inflammation and intercellular communication in shaping the immune microenvironment of intervertebral disc degeneration
Source: Front Immunol. 2025 Nov 19;16:1719293. doi: 10.3389/fimmu.2025.1719293 (PMC12672235; doi:10.3389/fimmu.2025.1719293)
Supplement: Supplementary file 1 [file DataSheet1.docx]

Supplementary Material

# Supplementary Tables

**Supplementary Table 1.** Summary of obtained datasets.

| Datasets | Platform | Tissue | Total samples | IDD Samples | Healthy Samples |
| --- | --- | --- | --- | --- | --- |
| GSE124272 | GPL21185 | Blood | 16 | 8 | 8 |
| GSE150408 | GPL21185 | Blood | 59 | 17 | 17 |
| GSE153761 | GPL22120 | Cartilage endplate in cervical disc | 6 | 3 | 3 |
| GSE244889 | GPL24676 | Nucleus pulposus tissue | 13 | 13 | 0 |

**Supplementary Table 2**. Genes selected by four machine learning algorithms.

| Model | Genes | Model | Genes | Model | Genes | Model | Genes |
| --- | --- | --- | --- | --- | --- | --- | --- |
| Lasso | FCGR1A | Neural network | UCHL1 | SVM-RFE | KLRK1 | RF | KLRK1 |
|  | MMP9 |  | CCR9 |  | HPGD |  | HPGD |
|  | AZU1 |  | KLRB1 |  | KLRB1 |  | KLRB1 |
|  | KLRK1 |  | MMP9 |  | MMP9 |  | UCHL1 |
|  | KLRB1 |  | MPO |  | KLRC3 |  | MMP9 |
|  | KLRC3 |  | KLRC3 |  | UCHL1 |  |  |
|  | UCHL1 |  | KLRF1 |  | ANKRD22 |  |  |
|  | ANKRD22 |  | HPGD |  | CCR9 |  |  |
|  | HPGD |  | ANKRD22 |  | AZU1 |  |  |
|  |  |  | CEACAM6 |  | KLRF1 |  |  |
|  |  |  |  |  | CEACAM6 |  |  |
|  |  |  |  |  | FCGR1A |  |  |
|  |  |  |  |  | ELANE |  |  |

# Supplementary Figures


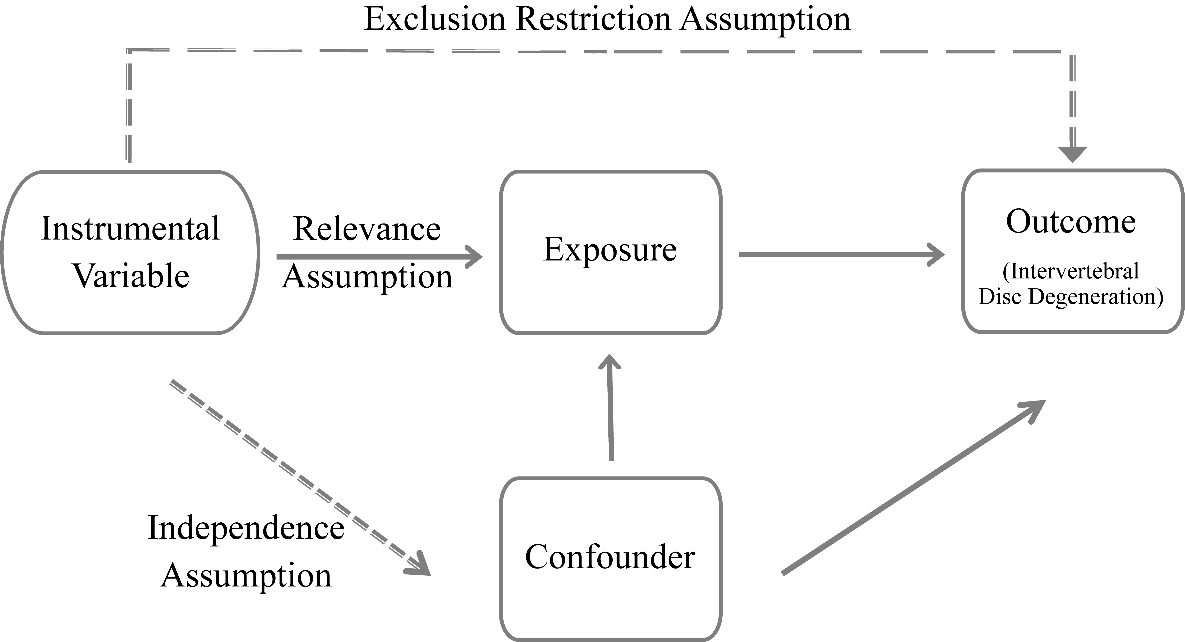


**Supplementary Figure 1.** MR Analysis Flowchart.


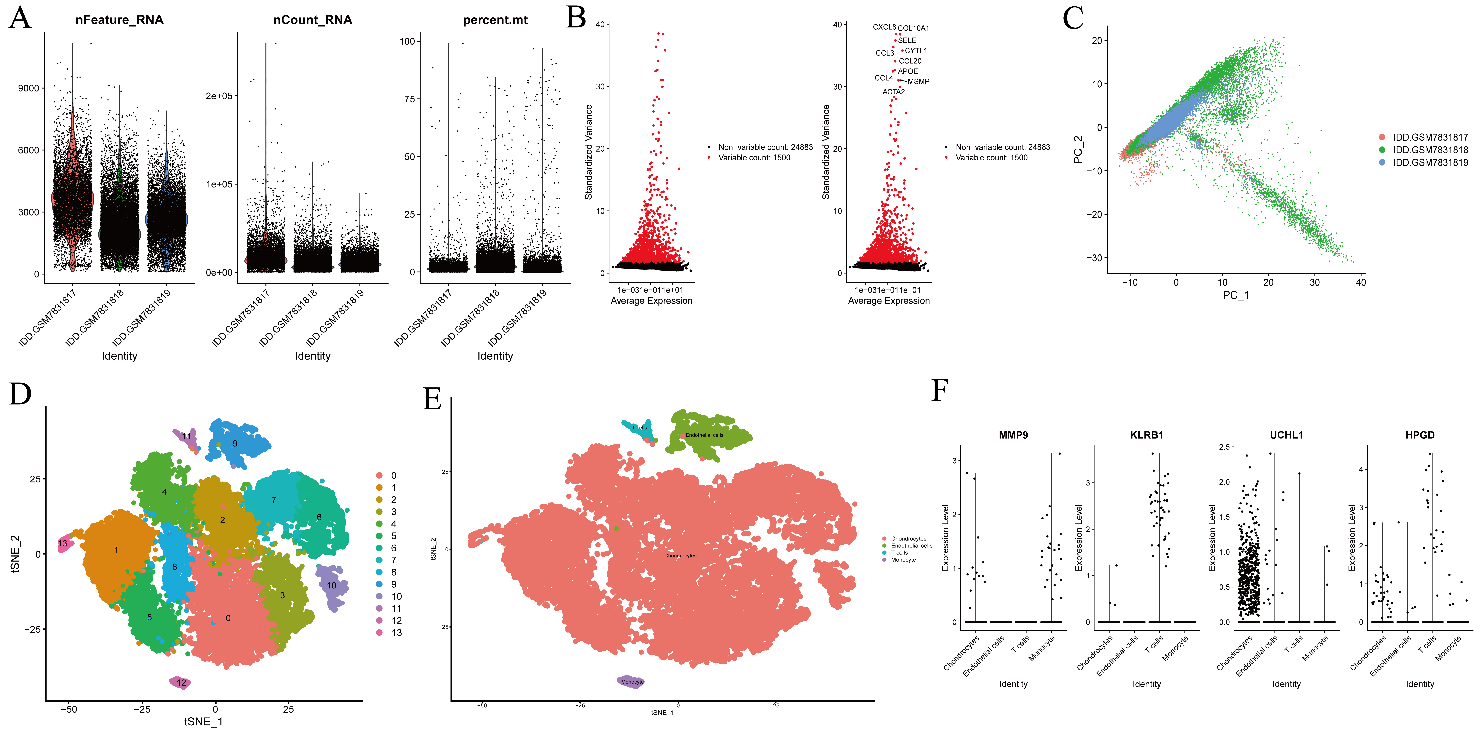


**Supplementary Figure 2.** Based on single-cell RNA sequencing (scRNA-seq) data, different annotated cell clusters in IDD samples were identified. (A) Quality control of scRNA-seq data; (B) Variance plot showing gene expression variability across all cells; (C) PCA showing clear separation of cells; (D) tSNE successfully classified cells into 14 subpopulations; (E) Annotation of all 4 cell clusters; (F) Bubble plot showing the expression of four core genes.
